# Reaching the hard-to-reach: a systematic review of strategies for improving health and medical research with socially disadvantaged groups

**DOI:** 10.1186/1471-2288-14-42

**Published:** 2014-03-25

**Authors:** Billie Bonevski, Madeleine Randell, Chris Paul, Kathy Chapman, Laura Twyman, Jamie Bryant, Irena Brozek, Clare Hughes

**Affiliations:** 1 School of Medicine & Public Health, Faculty of Health & Medicine, University of Newcastle, Callaghan, NSW, Australia; 2 Health Behaviour Research Group, Priority Research Centre for Health Behaviour, Faculty of Health & Medicine, University of Newcastle, Callaghan, NSW, Australia; 3 Cancer Council NSW, 153 Dowling Street, Woolloomooloo, Sydney, NSW, Australia; 4 School of Medicine & Public Health, Calvary Mater Hospital, University of Newcastle, Level 5, McAuley Building, Callaghan 2308, NSW, Australia

**Keywords:** Systematic review, Medical research, Vulnerable groups

## Abstract

**Background:**

This study aims to review the literature regarding the barriers to sampling, recruitment, participation, and retention of members of socioeconomically disadvantaged groups in health research and strategies for increasing the amount of health research conducted with socially disadvantaged groups.

**Methods:**

A systematic review with narrative synthesis was conducted. Searches of electronic databases Medline, PsychInfo, EMBASE, Social Science Index via Web of Knowledge and CINHAL were conducted for English language articles published up to May 2013. Qualitative and quantitative studies as well as literature reviews were included. Articles were included if they reported attempts to increase disadvantaged group participation in research, or the barriers to research with disadvantaged groups. Groups of interest were those described as socially, culturally or financially disadvantaged compared to the majority of society. Eligible articles were categorised according to five phases of research: 1) sampling, 2) recruitment and gaining consent, 3) data collection and measurement, 4) intervention delivery and uptake, and 5) retention and attrition.

**Results:**

In total, 116 papers from 115 studies met inclusion criteria and 31 previous literature reviews were included. A comprehensive summation of the major barriers to working with various disadvantaged groups is provided, along with proposed strategies for addressing each of the identified types of barriers. Most studies of strategies to address the barriers were of a descriptive nature and only nine studies reported the results of randomised trials.

**Conclusions:**

To tackle the challenges of research with socially disadvantaged groups, and increase their representation in health and medical research, researchers and research institutions need to acknowledge extended timeframes, plan for higher resourcing costs and operate via community partnerships.

## Background

The omission of groups of lower socioeconomic status from public health and medical research has been observed for some time regardless of type of research study [1]. In most Western developed countries white, middle class, highly educated males tend to be overrepresented in health and medical research and people from socially disadvantaged groups under-represented [1,2].

Failure to obtain medical research data which accurately reflects the breadth of the whole population poses a number of drawbacks including threats to external validity and ability to generalise [3], denying excluded groups from any health benefits of trial participation [4], inability to check the safety of health innovations with sub-groups in the population [5], and failing to identify groups that have the highest burden of illness and developing an understanding of why differences exist [6].

Researchers continue to struggle to access, engage and retain participants from socially disadvantaged groups [7], resulting in labels such as “hard-to-reach” or “hidden”. According to Sydor’s [8] definition that *“hard to reach populations are difficult for researchers to access”*, and Lambert and Wiebel’s [9] definition of hidden populations as *“those who are disadvantaged and disenfranchised: the homeless and transient, chronically mentally ill, high school drop-outs, criminal offenders, prostitutes, juvenile delinquents, gang members, runaways and other street people”,* socially disadvantaged groups are difficult for researchers to access cost-efficiently in large numbers necessary for statistically powerful study designs. There are many reasons why socially disadvantaged groups are not included in health and medical research. Understanding these factors is necessary for developing strategies to increase the level of involvement and participation in health and medical research for disadvantaged groups. This study aims to review the literature regarding the barriers to sampling, recruitment, participation, and retention of members of socially disadvantaged groups in health research and the strategies for overcoming the barriers that may help increase the amount of health research conducted with socially disadvantaged groups.

## Methods

### Search strategy

Searches of electronic databases Medline, PsychInfo, EMBASE, Social Science Index via Web of Knowledge and CINHAL were conducted for English language articles published up to May 2013. A wide-net search strategy involving combinations of the following keywords was initially conducted to capture as broad a sample of studies as possible: “difficult to reach” or “hard to reach” or “social disadvantage” and “health research”. An iterative process was used where more general searches were conducted initially, with papers identified informing subsequent targeted searches. A general internet search with Google Scholar also assisted in the search for grey literature. Free text searching was implemented using the following key words: ‘hard-to-reach’ , ‘difficult-to-reach’ , and ‘disadvantaged’ with ‘health’ and ‘health research’. In addition, manual checks of the reference lists of retrieved articles and citation searches were conducted.

### Selection criteria

Articles were included if they reported attempts to increase socially disadvantaged group participation in research, or barriers to conducting research with socially disadvantaged groups. Socially disadvantaged groups were defined as socially, culturally or financially disadvantaged compared to the majority of society, implying individual, environmental or social restrictions to their opportunities to participate in health research [8-10]. In order to capture a broad representation of evidence, qualitative, quantitative, mixed methods, case studies and literature reviews were included. Only studies that focused on health-related research were included.

Studies were excluded if they: described improving access for socially disadvantaged groups to health services rather than health research; were primary articles which had been cited in the previous literature reviews; focused on age (e.g., adolescents) or gender groups (e.g., females) without explicitly stating that the group was socially disadvantaged. Editorials and commentaries were also excluded.

### Data extraction

Articles obtained from the electronic database searches were assessed by one reviewer (MR) in two phases: 1) title and abstract review, and if it appeared relevant, 2) full text review.

Together with a second reviewer (BB) full text review of articles obtained was conducted. Uncertainty was resolved through consultation with the other reviewer. Two reviewers (BB and MR) extracted data on country, descriptors of the study’s target sample group, variables measured, study design, and key findings on a) the barriers to participation in health research; and b) strategies to improve participation were extracted and summarised.

The results of the included studies were further categorised according to five stages of a research study where representativeness may be threatened: 1) developing a sampling frame; 2) recruitment and gaining consent; 3) data collection and measurement; 4) intervention delivery and uptake (if applicable); and 5) retention and attrition. These stages were imposed by the study authors prior to data extraction.

### Assessment of risk of bias

Given the considerable heterogeneity of study types, a systematic scoring system for evaluating the methodological characteristics of individual studies or risk of bias was not applied. Instead, a graded system of ‘levels of evidence’ based on study design alone was used. A number of hierarchies of evidence exist [11-13] and were adapted for this study (refer to Table 1).

**Table 1:** Summary of the 116 included studies - study designs and level of evidence

**Level of evidence**	**Study type**	**N of studies**	**Reference**
Good	Randomised controlled trial (RCT)	9	[23,24,36,37,42,43,88,90],[106]
Fair	Non-RCT comparison of strategies or groups	14	[17,25,41,59,61,62,79,83],[104,109,110,116,126,128]
Poor	Descriptive study (with quantitative data, e.g., % of target group recruited, often single strategy pre-post)#	48	[14-16,18-22],[26,27,30,33,35,38-40],[44-47,49-51,53],[54,58,65-67];[71,75,76];[77], [81,84,85,87,91,95-97],[99,112,113,117,119,120,125,129]
Poor	Qualitative study (e.g., focus groups or interviews)	16	[29,32,60,64,68,72,73,86],[89,92,94,101,108,122,124,127]
Poor	Case studies without data	28	[28,31,34,48,52,55-57,63,69],[74,78,80,82,93,98,100,102],[103,105,107,111,114,115,118,121],[123]

#Descriptive studies are observational, with no comparisons made but data is reported (either increasing sample size, response rate, representativeness or acceptability).

## Results

**Figure 1:**
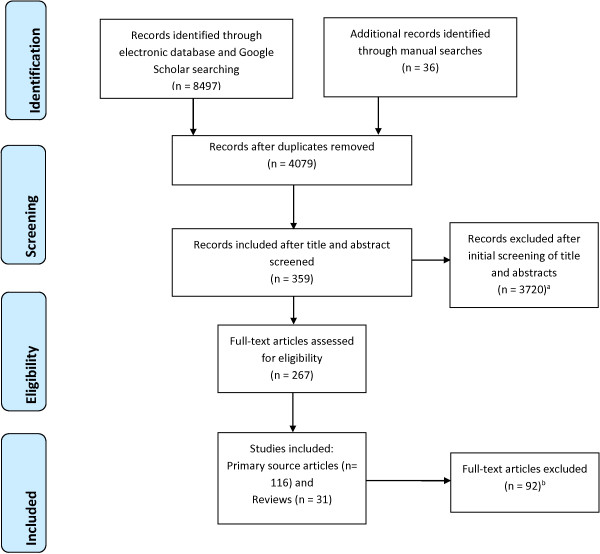
**Flow chart of included studies.** Footnote: ^a^ Did not report barriers or strategies to improve participation (n = 3118); Not socially disadvantaged group (n = 453); Focus on health service participation rather than health research participation (n = 91); Not English language (n = 58). ^b^ Focus on health service participation rather than health research participation (n = 53); Not socially disadvantaged group (n = 39).

The electronic searches yielded 8,497 potential articles and 36 articles were identified using additional search strategies. Following removal of duplicates, eligibility screening yielded 116 primary source papers from 115 studies (one study had two publications [14,15]) that met inclusion criteria (see Figure 1).

The majority of the included studies (n = 76) were based on research conducted in the United States of America (US) [16-91], 23 papers describing 22 studies were conducted in the United Kingdom (UK) [14,15,92-112], nine in Australia [113-121], four in Canada [122-125], two in Europe (Russia and Estonia [126] and Germany [127]), one in New Zealand [128], and one US-led study based in Mexico [129]. Table 2 summarises the socially disadvantaged groups targeted in the included studies. African American and diverse ethnic and racial groups (dominated by US based studies) were the most studied groups.

**Table 2:** Summary of socially disadvantaged groups in the 116 included studies

**Group**	**N of studies**	References*
Ethnic/racial groups (e.g. Latino, Hispanics, Asian, or mixed)	40	[14-16,18,20-22,29,38,42,44-46,56],[57,60-64,67,77,80,85-87,90,92-94],[101,103-105,107-112,127]
African American	19	[19,25,28,31,36,37,41,43],[44,47,50,57,58,70-73,75,81]
Substance abusers	14	[23,24,33,34,65-67,79,80,82],[83,95,98,126]
General - ‘vulnerable’, ‘minority’, ‘disadvantaged’	11	[30,32,48,51,52,72,97,78],[84,89,120]
Indigenous people	8	[113-115,117,118,121,125,128]
Low income, disadvantaged area	7	[54,64,96,99,106,108,119]
HIV (alone or with drug addiction or ethnic)	7	[35,64,67,76,100,124,129]
Low income rural	7	[49,68-71,74,88]
Gay, lesbian, bisexual and transgender (GLBT)	4	[38,51,83,102]
Low literacy group	4	[45,48,86,87]
Homeless people	4	[27,40,59,95]
High risk Youth	3	[39,55,66]
Survivors of violence	3	[17,26,53]
Sex workers	1	[122]
Mental illness	1	[40]
People with a disability	1	[116]

*Study may appear in more than one category if more than one population group was investigated.

The vast majority of articles describing strategies for improving representativeness were non-experimental descriptive surveys, qualitative studies or case studies (see Table 1). Only 7 articles reported the results of randomised controlled trials.

In addition, 31 previous reviews of related literature were identified and summarised in Table 3[130-158] including two papers which presented both original data as well as a literature review [14,34].

**Table 3:** Summary of previous published literature reviews (n = 31 reviews) of barriers and/or strategies to increase disadvantaged group representation in health research

**Author & Year**	**Type of review**	**Target group/s**	**Barrier/s addressed**	**Strategies reviewed**	**Main results & conclusions**
**Sampling**					
Marpsat & Razafindratsima [152]	Narrative with 60 included studies	General ‘hard-to-reach’	No sampling frame – difficulties sampling hidden groups and rare populations	1. Time-location sampling.	Each non-probability sampling method described advantages and disadvantages of each strategy presented with no preferences recommended.
				2. Respondent driven sampling.	
				3. Capture-recapture (or contact-re-contact).	
Malekinejad et al. [151]	Systematic review with quantitative analysis with 123 included studies from Africa, Asia, Europe, Latin America, Oceania	HIV related (injecting drug users, sex workers and homosexual men)	Sampling for HIV surveillance studies	Respondent drive sampling	All but 13 studies reached >90% of their intended sample size and only six failed to reach equilibrium for key demographics suggesting representativeness. The majority of studies used formative research, face-to-face interview formats, three referrals per participant, a single interview site for data collection, and incentives.
Aldana & Quintero [153]	Narrative review with 18 included studies	General ‘hard-to-reach’	Sampling for surveillance studies	1. Venue-based time-location sampling.	Advantages and disadvantages of each strategy discussed.
				2. Targeted sampling.	Targeted sampling which requires ethnographic data provides important qualitative information.
				3. Respondent driven sampling	Venue-based sampling allowed a systematic recruitment of participants and produced probability samples but only of the visits to the venues included in the sample.
					Respondent driven sampling based on social networks permitted calculation of population estimates.
Atkinson & Flint [150]	Narrative review with 22 included studies	General ‘hard-to-reach’	Sampling in general	Snowball sampling	Described advantages and disadvantages of snowball sampling with no comparison with other methods. Recommended particularly for qualitative research.
Peterson et al. [34]	Narrative review with 32 studies	Illicit drug users	Sampling following discharge from treatment	Targeted sampling (i.e., non-probability sampling method requiring identification of high yield locations).	Comparison with ‘in treatment’ samples suggested representative samples obtained using targeted sampling.
Faugier & Sargeant [154]	Narrative review with about 30 studies (number not reported, estimated from reference list)	Sex workers, HIV, substance abuse populations	Sampling issues	Non-random methods of sampling such as snowball sampling.	Snowball sampling, although not probability or random sampling, has been used for research with hard to reach groups.
Andresen et al. [138]	Narrative review with about 100 studies (number not reported, estimated from reference list)	‘Low-frequency’ populations in public health surveillance surveys	Sampling groups with small numbers in the population results in small samples of hard to reach groups in population surveys	Improving analysis using:	Discussed advantages and weaknesses of each strategy for increasing small-group participation in population surveys. Recommendations included:
				• Aggregating data – by location or time	Partnering with agencies (e.g., state hospital associations);
				• Spatial smoothing	Bilingual interviewers;
				• Small area estimation	Including non-random sampling methods to enhance samples;
				• Exact statistics	Changes in statistical methods.
				• Provider profiling methods	
				Using non-probability sampling strategies:	
				• Convenience sampling	
				• Snowball methods	
				• Publicly available or commercial lists	
				Improving recruitment methods:	
				• Extending survey modes (to include face to face)	
				• Staff training	
				• Incentives	
				• Involve community members	
				• Bilingual interviewers and surveys	
				Data collection methods:	
				• Use qualitative methods and participatory research	
				• Multi-method surveys	
Kalton [149]	Narrative and selective review with about 90 studies (number not reported, estimated from reference list)	Rare (low prevalence) populations	Sampling	Over-sampling	Options discussed include:
					• Disproportionate stratified sampling;
					• Two-phase sampling;
					• Use of multiple frames;
					• Multiplicity sampling;
					• Location sampling;
					• Panel surveys;
					• Use of multi-purpose surveys.
					Recommended use of more than one sampling method and tailoring based on study research question.
**Recruitment**					
Dhalla & Poole, [147]	Systematic review with 19 studies from OECD countries and 39 studies from non-OECD countries	Hard-to-reach participants for HIV vaccine trials	Low recruitment rates into HIV vaccine trials	Two types of participant motivation to participate in HIV vaccine trials: altruistic (social benefits) and personal benefits)	Motivators which may help develop and tailor recruitment strategies included: (altruistic) to protect partner/community/others; help stop spread of AIDS; help research; help find cure; (personal) protection from HIV; because friends are; to enjoy sex; monetary and non-cash incentives; doctor/military suggestion; personal recognition; free health care.
Swanson & Ward [131]	Systematic review with qualitative synthesis with 107 studies (about 50% were on the barriers to recruitment only)	American ethnic minorities	Recruitment into clinical trials	Effective and ineffective methods outlined	‘Effective’ methods for recruitment based on methodologically superior studies included:
					• Community partnerships
					• Community leaders
					• Community involvement in development
					• Formative qualitative research
					• Gifts and incentives
					• Provide transport
					• Community advisors
					• Sustainable interventions
					• Grants to health services
					• Establish networks of doctors
					• Cultural/family tailoring
					• Recruitment materials in target language
					• Tracking databases
					• Active recruitment with research staff to help completion of forms
					• Train staff in cultural issues
					• Employ community residents as part of research
UyBico et al. [141]	Systematic review with quantitative analysis and consideration of studies’ methodological quality with 20 included studies	Vulnerable populations	Recruitment into intervention health research	1. Social marketing (e.g. mass mailing, telephone calls, mass media).	Successful strategies included:
				2. Community outreach (via churches, community organisations, presentations/meetings, community events, door to door canvassing).	• Social marketing was successful in 44% of studies.
				3. Through health system (doctor referral, health centre recruitment, registry, patient records)	• Health system based strategies were successful in 40% of studies.
				4. Referrals (by friends/family, other research participants)	• Referrals strategies were successful in 35% of studies.
					• Community outreach was successful in 13% of studies
Lai et al. [142]	Systematic review with qualitative synthesis with 14 included studies	Under-represented populations	Recruitment into cancer clinical trials	1. Recruitment letters/flyers and telephone calls.	Only three studies reported efficacious strategies:
				2. Incentives and gifts.	• Media campaign (compared to clinic registry recruitment)
				3. Recruitment facilitators (doctors, insurers, businessmen and community organisations).	• Enhanced mailing process, church project sessions conducted by African Americans, letters and telephone reminders (compared to mailed survey only and telephone only)
					• Companies providing researchers with names and phone numbers (compared with employees actively signing up at work)
Howerton et al. [143]	Systematic review with 18 studies	Under-represented populations (recruitment to cancer clinical trial)	Clinician characteristics, attitudes and practices	11 clinician level promoters of recruitment	Clinician factors such as communication style, lack of trust of research, lack of awareness, logistics and cost are barriers to the recruitment of under-represented populations in cancer clinical trials. Addressing those barriers through incentives (e.g., extra staff), provider training, institutional affiliation, helps improve attitudes and recruitment.
Wendler et al. [146]	Systematic review with 20 studies with meta-analysis	Racial and ethics minorities	Participants’ willingness to participate and attitudes towards research	Changing attitudes to research versus other pragmatic barriers to research.	No differences in willingness (as measured by consent rates of racial minority groups versus non-minority Whites) to participate in research by racial group. Attitudes towards research were positive. Efforts should focus on improving access to research not changing participant attitudes.
Hussain-Gambles et al. [14,15]	Systematic and thematic review with about 38 studies (number not reported estimated from reference list)	Ethnic minority groups	Participant fear and mistrust, inappropriate exclusion criteria and study designs, costs to researchers, lack of ethnic staff, socio-cultural issues, cultural myths	N/A – review of barriers only	Under-representation caused by a combination of factors that need to be addressed. Potential strategies are discussed, but not included in the review (such as strategies to reduce fear and mistrust, education and training, community links and advocacy, outreach strategies, recruitment through primary health care) to address barriers.
Ford et al. [132]	Systematic review with 46 studies	Racial and ethnic minorities, older, rural and low socioeconomic status	Mistrust of research, perceived harms, costs, transport, lack of education, time, fear, family, provider attitudes and characteristics, communication, lack of protocols, religious/spiritual beliefs, low health literacy, culture	N/A – review of barriers only	Concluded under-represented groups face numerous barriers to participation in cancer-related trials.
Guiliano et al. [144]	Non-systematic narrative review	‘Minority’ groups	Structural, cultural and linguistic factors limiting participation in cancer research	N/A- review of barriers	Research where participants feel ownership, trust and receive results more likely to increase participation.
Miranda et al. [145]	Narrative review with about 48 studies (number not reported, estimated from reference list)	Low income Latinos	Lack of insurance, time, child care, and transport. Cultural barriers and beliefs in traditional non-medical healers.	1. Use of health services for recruitment.	Research needs to be culturally sensitive and to remove logistic barriers. Bilingual and bicultural staff should be part of the research team.
				2. Bilingual and bicultural staff.	
				3. Developing culturally sensitive research materials.	
Flory et al. [140]	Systematic review with 30 included studies	Low literacy	Participants limited understanding and literacy	1. Multi-media,	Extended discussion (educator or staff spending more time talking one-on-one to participants) was the most effective strategy according to methodologically superior studies.
				2. Enhanced’ consent forms,	
				3. Extended discussions	
				4. Test/feedback (quizzing participants about the information)	
				5. Miscellaneous	
Shavers-Hornaday et al. [139]	Non-systematic and narrative review with about 100 studies (number not reported, estimated from reference list)	African-Americans	Participant barriers (distrust, health care access and utilization, quality of care).	Outlined strategies based on 11 cancer trials that successfully recruited African/Americans	Effective recruitment strategies based on the results of 11 cancer trials that successfully recruited representative samples of African American include:
			Investigator barriers (low recruitment and retention, cost, relationships with minority health professionals)/		• An active commitment to recruiting African American subjects;
					• Community outreach programs and advertisements;
					• Involvement of local churches and community organizations;
					• Publicity campaigns directed at African Americans;
					• Participant logistics such as convenient testing times, transport, convenient location;
					• Use of incentives;
					• Use of African American role models;
					• Flexibility and willingness to change protocol
					• Use of lay health workers;
					• Door to door canvassing
Ndumele et al. [156]	Systematic review with 45 included studies	Minority populations with chronic disease	Recruitment into qualitative research	1. Health care setting	Based on 21 studies that provided data-based results: no discernible patterns of recruitment method that seemed associated with greater rates of participant recruitment.
				2. Community organisations	
				3. Electronic or mailed invitations	
				4. Media	
				5. Word of mouth	
				6. Incentives	
				7. Recruits from existing databases	
**Data collection & measurement**					
Hergenrather et al. [133]	Systematic review with qualitative synthesis with 31 included studies	Various vulnerable populations	Engaging communities and collecting data in acceptable methods	Photovoice (i.e., use of photographs to encourage group discussion).	Photovoice expanded representation and diversity of community members participating in health research.
Halcomb et al. [134]	Integrative review with about 40 studies (number not reported, estimated from reference list)	Culturally and linguistically diverse (CALD)	Challenges in focus group research with CALD populations	1. Involvement of key members of the target group;	Involvement of leader members of the target group; bilingual facilitators; consider particular CALD group as not all are the same are key considerations.
				2. Logistical convenience;	
				3. Physical environment should be considered;	
				4. Bilingual facilitators;	
				5. Consider particular CALD group as not all are the same (e.g., incentives not acceptable to some).	
**Intervention participation and fidelity**					
Sheridan et al. [130]	Systematic review with 38 included articles	Low health literacy populations	Populations with low health literacy and language difficulties	Design features of health information interventions.	Multiple strategies are required to improve availability of health information and intervention for low literacy/numeracy groups. Design features found to improve participant comprehension:
					• Presenting essential information first or on its own;
					• Presenting disease risk or treatment benefit information using the same denominators
					• Presenting numerical information in tables not text
					• Adding icon arrays to numerical information
					• Adding video to verbal narrative
Glazier et al. [157]	Systematic review with 17 included studies	Socially disadvantaged groups	Diabetes care interventions for socially disadvantaged groups	Patient, provider and health system interventions for diabetes	Positive intervention features were:
					• Cultural tailoring
					• Community or lay educators
					• One-on-one interventions
					• Behaviour related tasks
					• Feedback
					• High intensity and long duration
					Negative intervention features were:
					• Didactic teaching
					• Focus on increasing (diabetes) knowledge
**Across stages of research**					
Yancey et al. [148]	Systematic review with qualitative synthesis and consideration of studies’ methodological quality with 95 included studies	African American and American ethnic groups	Recruitment and retention in general	1. Community involvement in research.	Based on methodologically superior studies, strategies recommended to increase recruitment and retention include:
				2. Incentives and logistical aids (e.g., transport).	
				3. Cultural tailoring.	
					• Population based sampling strategies are unlikely to produce sufficient numbers
					• Personal contact and mass media were efficacious for recruitment
					• Non-restrictive eligibility criteria
					• Community involvement more critical to retention than recruitment
					• Timely incentive payments
					• Cultural tailoring
Johnson et al. [155]	Systematic review with 6 included studies (RCTs only)	African American	Recruitment and retention into genetic and genomic studies	1. Population strategies (phone, mail or postcard);	• Phone recruitment (63%-91%) significantly better than mail or postcard (3%-19%).
				2. community-based strategies (community engagement and partnerships);	• Community engagement produced mixed recruitment results (1% - 82%) with locals as recruiters important success factor.
				3. Incentives	• Only 1 trial of incentives found no effect on retention.
Wallace & Bartlett [158]	Narrative review with about 38 studies (number not reported, estimated from reference list)	African American and Hispanic girls and women	Recruitment and retention	Recruitment:	Recommended using all of the strategies outlined.
				• Building trust	
				• Familiarity and visibility	
				• Racial and ethnic concordance	
				• Convenience	
				Retention:	
				• Provide transport	
				• Language, literacy and culturally appropriate,	
				• Emphasising safety	
				• Flexibility	
				• Incentives	
				• Regular communication	
				• Veracity	
Grove et al. [135]	Narrative review with 20 studies	Indigenous Australians	Recruitment and retention in longitudinal research	Community participation (and ownership), developing relationships.	Three studies reported successful recruitment and retention using community participation strategies.
Booth et al. [136]	Non-systematic (selective) narrative review with about 13 studies (number not reported, estimated from reference list)	Homeless people	Sampling and data collection, ethical and fieldwork issues		Key recommendations were:
				1. Convenience (non-random) sampling.	• Use common sense to enhance practicality and reduce burden on participants;
				2. Building trust.	• Cultural sensitivity;
				3. Simple consent materials.	• Take non-threatening approach;
				4. Incentives to participate.	• Appropriate language;
				5. Cultural sensitivity.	• ‘Hanging out’ with target group;
				6. Using a non-threatening approach.	• Provide feedback;
				7. Providing feedback.	• Note style of clothing;
					• Use informants and service providers;
					• Incentives
Mathers & Cramer [137]	Narrative review with about 28 studies (number not reported, estimated from reference list)	Gay, Lesbian, Bisexual, Transgender (GLBT)	Recruitment & data collection: difficulties identifying ‘hidden’ samples. Researchers as ‘outsiders’. Researcher verbal and non verbal cues influencing survey responses.	Web and videoconferencing	Web and videoconferencing are private and non-threatening for hidden populations and should provide more accurate data.

### Developing a sampling frame

#### Barriers

Of the 31 literature reviews, 10 focused on sampling [34,136-138,149-154] and 33 primary source articles on sampling issues were found [32-34,38,44,47,51,53,54,57],[60-62,67,74,77-79,81,84,91,95],[97,102-104,113,116,118,122,126,128] (see Table 4). Random population samples are often insufficient to accumulate large enough samples of hard-to-reach groups.

**Table 4:** **Summary of the results from ‘ *sampling *’ studies included in the review (n = 33 studies)**

**Barriers**	**Strategies**
Difficult to locate or reach and access groups (e.g., homeless people living on the streets)	• Snowball/social network or respondent-driven recruitment [33,57,60,62,79,84,102-104,116],[118,126,129]
Frequent change of address or self-identifying, (e.g. GLBT) results in no sampling frame.	• Time-space sampling [38,104,113]
	• Targeted sampling [34,79]
	• Capture-recapture [95]
	• Adaptive sampling [53]
	• Partnerships with community groups [32,40,44,47,54,60-62,67,74],[103,116,118,122,128]
Low prevalence in population (e.g., Aboriginal people).	• Combination of various data sources as a novel methodology to avoid sampling [62,97] or supplementing with additional data (e.g. from qualitative research) [44,47,54,60,67,103,113,122],[128]
	• Statistical analysis techniques to population survey data for low-frequency samples [91]
	• Internet samples [51]

This barrier was reported across study types, from large scale population health surveys to intervention trials. Some studies identified difficulties sampling groups defined as “hidden populations” consisting mostly of people who do not want to be identified such as people who use illegal substances or self-identified groups such as homosexual people, others described difficulties sampling groups with low numbers in the population such as Indigenous people. As a result population based probability sampling tends to be a time and cost inefficient strategy for sampling socially disadvantaged groups.

#### Strategies to improve sampling

##### Non-probability sampling

A number of alternatives to random probability sampling were described (see Table 4) including snowball/social network or respondent-driven recruitment, venue based time- location sampling, targeted sampling, capture-recapture, adaptive sampling and oversampling of low prevalence population sub-groups [33,34,38,53,57,79,84,95],[102,104,113,126,129].

Except for oversampling methods for low prevalence populations [91,149], all of the other sampling strategies require formative research to identify venues (places), times, and contact persons to develop a targeted sampling frame for the group of interest which may impose significant time and cost to the research. Some strategies such as snowball and respondent-driven sampling involve referral chains of sampling. Selection bias and gatekeeper bias which limit validity of the sample are the primary limitations of these strategies. While these issues may not be problematic for studies which do not require representativeness for generalizability, such as qualitative research, they do have limited use in quantitative research.

##### Sampling through community organisations

One option for creating a sampling frame for specific socially disadvantaged groups is to collaborate with community organisations with access to those groups and to draw a convenience sample through that organisation [44,47,54,60-62,67,74,103,116],[118,122,128,136]. Benoit et al. [122] variously defined community group as any group with high access to the target population and partnerships have taken three main forms: a) the community group helping researchers gain access to an otherwise hard-to-reach group; b) a reciprocal relationship in which community members and researchers have knowledge and learn from the other; and c) community-initiated research projects that seek academic partnerships and use the outcomes to direct policy and program delivery. While this form of convenience sampling may not be representative of the general target group, it presents pragmatic advantages for sampling large numbers of members of socially disadvantaged groups.

##### Combinations of sampling strategies

Fifteen studies described the use of a combination of sampling strategies [32,34,44,47,54,60,61,67],[74,103,104,113,116,122,128]. For example, Shedlin et al. [60] used snowball sampling within community groups. As most reports were case-studies it is not possible to compare the effectiveness of combined approaches.

##### Comparisons of strategies

A number of studies provided comparisons of different sampling strategies such as respondent driven sampling compared with targeted sampling finding that in most cases these sampling approaches produced similar sample size and representativeness, however there were differences in costs. Platt et al. [126] compared respondent driven sampling with snowball sampling to reach high risk HIV participants and found that although snowball sampling was more costly, it resulted in greater participant numbers. Keyzer et al. [62] found that direct mail, community outreach (including presentations and visits to churches and community centres) and recruitment through a health education council were the most cost-effective strategies for recruiting minority groups, while use of mass media and advertising was a high-cost low-yield strategy. In New Zealand, Mhurchu et al. [128] trialled three strategies for capturing Maori and Pacific Islander participants in their research of a tailored nutrition intervention on supermarket purchases; a mail-out to supermarket customers, approaching Maori and Pacific community groups located close to the supermarkets and ‘in-store’ recruitment where recruiters approached Maori and Pacific customers as they entered the supermarket. While the mail-out resulted in the highest overall number of participants, only 11% were Maori or Pacific Islander. In comparison, community and in-store strategies recruited the lowest numbers overall, but 96% of participants were Maori or Pacific Islander.

##### Other strategies

Dowrick et al. [97] suggest use of multiple sources of secondary and primary data, such as previous published data and qualitative interviews, as a strategy to overcome sampling difficulties in a study to assess mental health needs of hard to reach groups. This approach has limited applicability and cannot be used for prevalence surveys or intervention trials where contact with individual participants is required. Mathews & Cramer [137] suggested the use of the internet to identify and sample the “hidden” population of gay, lesbian, bisexual and transgender (GLBT) and Mathy et al. [51] described sampling methods over the internet for quantitative and qualitative research. No empirical evidence of effectiveness is presented.

### Recruitment and gaining consent

#### Barriers

Most of the literature reviews (20/31) discussed problems and solutions to recruiting socially disadvantaged populations into health research [14,15,131,132,134-148,156,158]. In addition, 58 primary source articles considered issues relating to low recruitment rates [16,18,21,22,26,28,30,31],[35-37,40-43,45,46,50,51,55,56],[60,64,65,70-73,75,77,78,80-83],[85,86,89,92-94,96,99-101,105,106],[109,112-115,119,123-125,127] (see Table 5).

**Table 5:** **Summary of the results from ‘ *recruitment *’ studies included in the review (n = 58 studies)**

**Barriers**	**Strategies**
Lack of trust in research/research team or uncertainty regarding how survey results will be used	• Community-driven research [44,56,70,71,75,114] and community partnerships [18,28,75,77,78,80,93,94],[100,101,113,115].
Fear of authority	• Peer or known recruiters [21,28,40,74,82,96,113,115],[105,121,123,124].
Perceived harms of research	• Sensitive wording: “study”, “conversation” and “dialogue” instead of “investigation”, “research” and “interview” [26,47,60,92].
Mistreatment and exploitation	• Use of ‘hand-written’ envelopes (vs. printed) [106]*
No benefits for participation (i.e., ‘fly in, fly out’ research)	• Enlisting community leaders (60, 113,114,115,127].
	• Commitment to “give back” to the community through sustainable interventions [31,94,114,115] or reciprocal benefits [64,74,101] or if not resourced to provide intervention, provide links to services [118] or minimal intervention controls [44].
	• Shared data ownership and publication [114,118]
	• Gifts with project logo [18,30,92-94,118,127] and incentives [42,47,119,123].
	• Thank you and award ceremonies and project feedback [114,118].
	• Emphasising potential benefits [74].
	• Improved communication and culturally relevant education materials [32].
Lack of education/awareness re research or health promotion/low health literacy, difficulties understanding consent and what the study is about	• Utilising appropriate media (print vs. TV vs. online) [18,25,41,56,65,85,87,119]; mass media [61,62,72,81] or social marketing strategies [37,47,72,83].
	• Provision of participant feedback regarding the research outcomes [30,115].
	• Public information sessions [47,116].
	• Simplified consent forms – large font, plain language, shorter sentences, in respondents language, ensure translation makes sense, wide margins, shorter paragraphs [45,86,114].
	• Bilingual recruiters and materials [18,56,85,92,104,112]
Cultural beliefs, gender roles/age related issues	• Cultural competence skills of research team/well trained research staff [16,22,30,56,63,101,104,118]
	• Culturally targeted media [41,72,113,115]
	• Mindful different cultures require different strategies [16,43,63,67,93,94,103,118],[125].
	• Recruitment strategies adapted to local conditions for a community-specific approach [16,63,85,86,93,113,125].
Gatekeepers (therefore patients/community are not aware of research): doctors or nurses who do not approach minority participants, high turnover of staff limits relationships	• Work with gatekeepers [14,15,100,103,123], employ locals as staff [22,55,93,113,118].
Doctor poor communication methods	• Ensure appropriate authorities are consulted [113,114].
Rigid exclusive eligibility criteria	• Patient education materials [32].
	• Financial incentives for recruitment partners to employ support staff to recruit [32,44,143,125].
	• Flexible eligibility criteria [35,50].
Stigma/fear of exposure	• Online focus group and interview research [51,52] or video recruitment [46].
	• Community advisory group [28,47,100,113,118].
Low response rates in general	• Multiple (>6) contact attempts [40,66,81,99].
	• Toll-free number [61,70] or follow-up a mail survey with a telephone survey of non-responders [106].
	• Through doctors/health services [85,93,103,123].
	• Outreach/home visits [21,25,99].
	• Text messaging [65].
	• Incentives [18,30,40,42]*,[43]*,[70,81,92-94,123,127].
	• Recruitment letters: An advance letter (prior to a mailed survey) [36]* or culturally framed letter [43]*.
	• Two stage recruitment 1) to a low commitment survey then 2) to the trial [83].
	• Assistance with transport or child care [30,73].
	• Shorter surveys [106].
	• Develop a registry with interested people [25].

*Indicates good evidence from randomised controlled trial (see also Table 1).

Reasons provided for low response rates in research with socially disadvantaged groups included: mistrust in research or researchers, particularly amongst African Americans and Indigenous populations who had a history of being mistreated in medical research; fear of authority; and perceptions that participation presented no personal benefit to them or their community and may cause potential harm, stigma, mistreatment or exploitation. Similarly members of some groups may fail to participate in research out of fear of being publically exposed, particularly if they engaged in illegal behaviours such as prostitution, gambling or illicit drug use or are socially stigmatised, such as people with HIV or AIDS or people who are GLBT. One review however found that willingness to participate in research was as high in racial and ethnic minority groups as it was in Caucasian participants [146], suggesting that factors other than participant attitudes or beliefs play a role in limiting health research participation.

Other barriers to the recruitment of vulnerable populations included cultural beliefs prohibiting participation; age and gender issues whereby in some cultures discussing health issues is viewed as ‘sensitive’ , particularly if female, elderly or young; and a lack of awareness of health research or education about participation in health research. Low literacy affecting ability to provide informed consent was also described as a barrier to recruitment.

Gatekeepers who restrict access to health research have been identified by some studies as a barrier for research participation. Some health professionals, who have the opportunity to encourage research participation have been found to fail to do so due to paternalistic beliefs that people in lower socioeconomic groups don’t have the time, interest or ability to participate, or have poor communication skills.

Participant lack of understanding of the research information, process or significance was reported as barriers to gaining consent. In one case, participants not understanding the need for “random” sampling was reported as a barrier to gaining consent, since potential participants believed that those who needed the research should be approached, not randomly sampled [113]. Other papers highlighted the restrictive nature of some eligibility criteria (such as language or comorbidity-related restrictions) which excluded socially disadvantaged groups, particularly in clinical trials [14,15,50].

#### Improving response rates

##### Community-research partnerships

In order to address some of the recruitment barriers relating to mistrust or fear of research, and gatekeepers impeding recruitment, 25 studies [18,21,22,31,40,55,56,60],[63,70,77,78,80,82,93,94],[96,100,101,105,113-115,124,125] and eight reviews [131,135,136,141,144,145] suggested that community groups be involved in the research and recruitment process. This may be particularly effective for communities that have hierarchical structures such as Australian Aboriginal communities who look to their elders to provide leadership. Some reports have suggested that engagement of local peer or known community members as ‘recruiters’ will increase trust and response rates [21,40,55,82,96,105,121,123],[124]. Similarly, the use of community advisory groups is likely to be beneficial and increase the perception that the research is community-driven and responsive [28,47,100,113,118,135]. Germino et al. [28] used a comprehensive community based approach to recruit a representative sample of African American cancer survivors. The approach was designed to address recruitment barriers of mistrust and enhance familiarity.

They engaged a number of community groups, for promotion and education about the research including ‘cultural brokers’ to liaise between participants and researchers. They reported high recruitment and retention rates.

These community based strategies offer shared ownership of the data and publications produced as a result of the research; ensuring that the research will provide either sustainable programs beyond the life of the research project, or links to services and resources. Providing gifts, financial incentives or thank you awards and ceremonies which include feedback to the community about the outcomes of the research were also presented as important components of community-based recruitment [18,30,92-94,127,158].

In a review of recruitment strategies for clinical trials with minority groups by UyBico et al. [141], community organisation-based recruitment was found to be the least effective form when compared to social marketing, use of health services and referral based recruitment. Similarly, Martin et al. [21] found that despite extensive community consultation and use of community based facilities and bilingual recruitment materials, low recruitment of Mexican American participants persisted. Recruitment increased once local Spanish speaking workers were engaged to conduct outreach recruitment.

##### Use of media and social marketing

Eleven studies reported the use of media and social marketing techniques tailored to the target audience [18,45,46,65,72,73,81,85],[86,99,119]. However, these studies fail to provide a guide as to which medium would be most effective with different target groups and instead suggest that formative research should determine the most appropriate strategy. UyBico et al’s [141] review found social marketing (defined as mass media, mass mailings and mass telephone calls) to be the most effective recruitment strategy for minority groups into clinical trials, compared with health provider recruitment and community organisation-based recruitment.

A key component which is common across studies recommending the use of different recruitment channels is the need for the recruitment channel to be culturally and linguistically appropriate [16,45,71,86,92]. This can address both barriers of lack of awareness of health research, and cultural barriers. Studies also highlight the need to educate research staff to ensure cultural competencies and understanding which are likely to enhance response rates [22,30].

##### Strategies to encourage gatekeeper support

Strategies for addressing the barrier of gatekeepers include employing gatekeepers as project recruitment officers and involving them in the research [22,93,100,113,118]; ensuring that community authorities are informed about the research and adequately consulted [113,114]; and paying health professionals through financial incentives to assist with recruitment [125,143]. Loftin et al. [71] reported slow recruitment and low response rates in a study using primary health care provider identification and recruitment of African Americans into diabetes research. One review suggested developing patient materials that can be distributed directly to potential participants of clinical trials thus overcoming the barriers of clinicians being too busy to recruit or having poor communication skills [143]. Mathy et al. [51] compared an internet derived sample with a Gallup poll sample of the US general population. They found the samples equivalent in educational distribution and geographical location (rural and urban), and the internet sample reached more representatives of lower income and ethnic diversity.

##### Comparison studies

Four of the nine RCTs examined the effectiveness of recruitment strategies [36,42,43,106], as did three non-RCT comparison studies [41,75,83]. In a RCT by Satia et al. [43], potential African American participants were randomly assigned to receive either generic or culturally sensitive invitation letters. Within each letter group participants were randomly assigned to receive a small incentive (a telephone card worth USD $3.60). While the overall response rate was low (17.5%), it was significantly higher for those receiving the incentive (23.9%) compared with those not receiving an incentive (15.8%). There was no difference in response rates between those receiving the generic invitation letter and the ‘culturally sensitive’ letter [43]. Maxwell et al. [42] trialled three incentive conditions (no incentive, USD $5 cash or $20 promise upon completion) to increase response rates to a baseline survey amongst racially diverse groups (Latino, Asian and African American). No differences were found and response rates were low (28%-37%). Another RCT assessed whether an advance letter mailed out two weeks prior to a mailed survey would enhance response rates among African American participants compared with White American participants [36]. Statistically significant differences were found for white American participants only illustrating how a strategy can potentially contribute to disparities. In a RCT of hand-written envelopes compared with printed envelopes to increase survey response rates in a socioeconomically disadvantaged area, Choudhury et al. [106] found that the hand-written envelopes resulted in slightly but not significantly higher response rate (17% vs. 14%). Using ‘debriefing questionnaires’ to glean interviewer experiences of recruiting subjects using three different methods, McLean & Campbell [109] found that local advertisements and use of media recruited the highest number of white English participants, interpersonal contacts increased recruitment of Pakistani-Kashmiri subjects and organisational contacts recruited the most African-Caribbean subjects. All approaches included financial incentives for participation. Oakley et al. [112] compared the cost of using interpreters to increase recruitment of non-English speaking women. Costs per person recruited were higher for women who needed an interpreter (average of £135) compared with for those who did not (£80).

One unique study [25] sought to develop an African American ‘health research registry’ for easier future recruitment and engagement with research. The study compared direct recruitment methods (using existing study sample databases; public databases; community outreach) with indirect methods (radio, internet and email). The study concluded that all strategies were needed to recruit a representative sample into the registry because some strategies were more successful with sub-groups (e.g., email and internet methods recruited younger participants).

##### Other strategies

A number of reports have suggested that study inclusion criteria need to be broadened in order to increase the representativeness of samples, particularly in relation to comorbidities [35,50]. Adams-Campbell [50] suggested that investigators incorporate examination of comorbidities into clinical trial study protocols.

A novel suggestion to tackle the barrier of uncertainty about ‘random’ sampling is to include others within the participants’ immediate family or community in data collection [113,135], but only to use the data from the individual that was randomly sampled. Clearly this approach has ethical, resource and cost implications.

Sutherland et al. [26] described a range of personalised strategies to aid recruitment of abused rural women into sensitive research about the risk of sexually transmitted disease.

They found that personalised approaches like knitting together, and changing terminology to promote trusting relationships (such as “the nurses doing the research” instead of “the researchers”) boosted recruitment rates [26].

### Data collection and measurement

#### Barriers

In total, 29 studies [45,48,49,52,54-56,59,60,63],[68,74,85,90,96-98,100,105,106],[110,111,113-115,117,118,120,124] and seven reviews [133-138,140] explored barriers and solutions for collecting research data with participants from socially disadvantaged groups (see Table 6).

**Table 6:** **Summary of the results from ‘ *data collection *’ studies included in the review (n = 26 studies)**

**Barriers**	**Strategies**
Language or literacy problems	• Measures in other languages or bilingual interviewers [45,63,85,90,96,100,111,113].
	• Use of multi-media [48,106], or computer data collection [48,59,120].
	• Avoid self-administered surveys [117] use of objective data [97,117].
	• Short surveys [106].
Lack of landline telephone (for population based telephone surveys) or highly mobile population	• Mailed survey instead of telephone [90]*.
	• Supplement telephone with face-to-face surveys [49,110].
	• Online surveying [52,54,68].
	• Use of objective (e.g. tobacco sales) data instead of self-report [97,117].
	• Flexible data collection [60,113,114].
Mistrust of researchers and the use of the data	• Culturally trained interviewers [56,111] or locals [55,74,96,98,105,115,118,124].
	• Need to pilot test measures [100,114,115].

*Indicates good evidence from randomised controlled trial (see also Table 1).

The need for complete and representative data from health research studies is common across study types but is particularly important in surveys. Language, lack of education and low literacy barriers may prohibit the collection of certain types of data such as self-administered survey data. Similarly, a barrier which is specific to telephone-based data collection is the lack of landline telephones amongst disadvantaged groups, limiting their inclusion in epidemiological and population-based research. Shebl et al. [49], for example found significant differences between those with or without landline telephones in race/ethnicity, health care access, insurance coverage and several types of health behaviours including smoking status and cancer screening behaviours. The resulting bias suggests that population-wide surveys utilising only landline telephone surveying techniques are not providing accurate estimates of health behaviours. Finally suspicion regarding the use of the data collected and mistrust of researchers were identified as barriers.

#### Improving data collection and measurement methods

##### Inclusive language and methods

One of the most common strategies to improve inclusion of linguistically diverse or low literacy groups in health research is to simplify the reading age of the study materials or to translate materials into other more common languages [45,63,96,113] and use bilingual research assistants [45,85,90,100,111]. A number of studies highlighted the need for culturally trained and skilled field-workers [56,111,140] or employing locals or peers to conduct field work [55,74,96,98,105,115,118]. Use of “insiders” (peer or local researchers) offers the added advantage of addressing any researcher mistrust or suspicion [105] as well as building the capacity of the community or organisation in conducting research. One method of data collection called Photovoice allows participants to use photos and pictures to respond to spoken questions or scenarios and to tell a ‘story’ [133]. Photovoice has been used with Australian Aboriginal communities where the telling of stories is often through paintings and art. Its use is limited as an exploratory qualitative tool and does not provide large-scale quantitative data.

##### Flexible data collection methods

In order to address barriers to socially disadvantaged groups participating in telephone surveys, a number of studies have outlined the need for flexibility and tailoring of data collection methods to participant circumstances [60,113,114]. If participants cannot be reached by telephone data collection, telephone surveys could be supplemented by face-to-face door knock interviews [49] or online surveys [52]. Allison et al. [110] caution that supplementing postal questionnaires with face to face interviews may not produce responses with equivalence as comparisons of these two modes of survey delivery resulted in very low kappa agreement scores for some items. Working with Australian Aboriginal communities Couzos et al. [114] proposed a ‘community-controlled’ research process including data collection and management which was flexible and involved locals employed to administer the research protocol.

Thomas et al. [117] has suggested that self-administered surveys should be avoided if possible with disadvantaged groups where it is difficult to collect data directly from the individual, and instead, community-wide objective data be used. These authors give the example of tobacco and alcohol sales data from Australian Aboriginal communities. This measure is only a proxy to behavioural measures and provides limited data about how the tobacco or alcohol might have been used and by whom. However, in some cases the objective data could be supplemented with smaller targeted qualitative research using methods such as Photovoice ([133]: see above) to gain a more complete set of information about a health concern or health behaviour. Similarly, Dowrick et al. [97] suggest using secondary data sources supplemented with small qualitative data collection as measurement tools in studies assessing health service needs with socially disadvantaged groups.

##### Use of technology to gather data

Five studies suggested using the internet or other technology-based strategies to collect data from socially disadvantaged groups such as those living in rural areas [48,52,54,68,120].

Hahn and Cella [48] described the acceptability of a touchscreen computer delivered health survey with patients categorised with high or low literacy ability and found that acceptability was high across both groups. Almost all patients (98%) felt the touchscreen survey was easy or very easy to use.

##### Pilot testing measures

Hing et al. [115] and Couzos et al. [114] highlight the importance of involving local community partners in the development of research protocols and materials when working with Australian Indigenous communities and the importance of extensive pilot testing of materials [100].

##### Comparison studies

One randomised trial of survey methods was included in the review [90]. In this trial, Ngo-Metzer et al. [90] compared data quality (response rates, missing data, internal consistency reliability and non-response bias) following a mailed health survey compared to one delivered by telephone with Asian American participants. The surveys were provided in both English and the target language (either printed or through bilingual interviews). No differences in reliability or non-response bias were found. The telephone interview resulted in a significantly higher response rate (75% versus 59%) and fewer missing items (1.67 versus 4.14) [90]. In a pre- and post-comparison study, Choudhury et al. [106] found that shortening a 12-page questionnaire to two pages significantly increased the response rate (37% versus 12%) in a study with respondents from economically deprived multicultural areas. For assessing substance abuse, Alemagno et al. [59] compared a telephone-based interactive voice response system to a face to face interview and found high reliability (% agreement = 80-95% and test-retest kappa = 0.65 – 0.91) and validity of the computer assisted telephone method (78% agreement with biochemical measure of substance use). The computerised voice telephone system offered participants anonymity [59].

### Intervention delivery and uptake

#### Barriers

Thirty six articles [19,20,27,29,31,35,37,40],[52,55,56,58,64,67-69,71,73],[77,78,80,82,85,87,88,105],[107,108,114,118,122,124] and five reviews [130-132,148,157] considered intervention fidelity (see Table 7).

**Table 7:** **Summary of the results from ‘ *intervention fidelity *’ studies included in the review (n = 33 studies)**

**Barriers**	**Strategies**
Concerns regarding: Randomisation, i.e., not getting treatment Loss of control (re: allocation) Mistrust	• Ensuring minimal standard of care for control group or minimal intervention for controls [29,40] or alternatives to randomised controlled trials [27].
	• Use trusted sources of information (e.g. doctor or family) [87].
	• Participant education regarding the benefits of randomisation [73].
	• Including peers and locals in intervention delivery [20,55,58,82,105,108,124].
Health intervention not culturally appropriate (with community values and beliefs)	• Community involvement in development, action research method or ‘cultural immersion’ [27,29,31,35,56,67,70,71],[74,77,80,85,107,108,113,114],[118,122].
	• Culturally tailored programs [19,29,52,58,68-70,85,88,118].
Distance for delivery of intervention (rural groups)	• Group-delivered (one-on-one) telephone intervention [88].
Negative framing of health information, emphasis on disparities	• Positive and progress emphasised in health information [37]*.

*Indicates good evidence from randomised controlled trial (see also Table 1).

Concerns about randomisation - that some members of the community will not receive what may be a beneficial intervention - has threatened the implementation of intervention trials in some communities [40,148]. In some cases the concern has been about the loss of control over deciding who receives an intervention and who does not [132]. These threats could result in contamination if intervention group participants or those conducting the randomisation offer the intervention to control group participants.

Equally of concern are interventions that do not align with the perceived needs or priorities of the target group or interventions (in terms of both content and delivery) which are not acceptable, feasible and culturally appropriate to community values and beliefs [71,80].

#### Improving intervention participation and fidelity

##### Alternative methodologies and study designs

Alternative study designs to the classic RCT have been proposed that may be more acceptable [27,131] such as multiple baseline designs, stepped wedge designs and wait-list control groups where the intervention is delivered to all groups at different times [29].

Yancey [148] outlines other designs whereby the control group either receive an alternative treatment or they receive the intervention after the trial is over (a waitlist control). Similarly, Hough et al. [40] randomised homeless people to four conditions; each providing interventions that were greater than the services participants were receiving before the study. Yancey et al. [148] and Woods et al. [73] also highlight the importance of educating participants of the need for randomisation to enhance understanding and ensuring that the control group receive, at the very least, usual care and that they are not having health care denied them.

##### Community/participant involvement in intervention design

Seventeen studies [27,31,35,56,67,70,71,74],[77,80,85,107,108,113,114,118],[122] suggested community involvement in the design of health interventions as a means of avoiding the barrier of culturally inappropriate interventions. While the opportunity to design pharmacological and clinical medical interventions together with potential participants is very limited, it is a strategy that is particularly relevant to public health or behavioural interventions. Involving the target group in intervention design for public health interventions and careful inclusive use of formative research to ensure that both the content and delivery of the intervention is acceptable to the target group can increase likelihood of uptake [70,80,113,114,118].

To design culturally appropriate smoking cessation interventions for American Indian communities, Fu et al. [29] conducted focus groups. Participants reported that the following features of the intervention were important to them: programs led by American Indians, opportunity to link with other American Indians interested in quitting, free nicotine replacement therapy, incentives, and culturally specific program components such as American Indian images, education on traditional tobacco use, messages that value family and include narratives or stories [29].

Ammerman et al. [31] described the development of a culturally appropriate intervention which was based on theories of sustainability and diffusion. This is viewed as an attempt to “give back” to participating communities through sustainable interventions which are adopted beyond the life of the research [31,78,114] and which build capacity within communities to address the health issue independently of the research [56,124].

Modifying interventions implemented in general populations so they are better tailored to disadvantaged groups such as low literacy or rural groups [19,52,58,68,69,87,130,157] has been suggested. Other studies emphasise the benefits of including community members, locals or peers as intervention delivery agents for improving compliance to the intervention [20,55,58,82,105,108,124,157]. For example, Hughes et al. [82] outlines a peer educator participant-driven intervention for injecting drug users as an ethical public health model. Similarly, Rothschild et al. [20] employed bilingual Mexican American Community Health Workers from local neighbourhoods as culturally competent peer interventionists in their trial of a diabetes management intervention for Mexican Americans and reported high intervention participation and fidelity.

##### Comparison studies

Two RCTs of intervention strategies tailored for disadvantaged groups were included in the review. In a blinded randomised trial, Nicholson et al. [37] compared emotional and behavioural responses to four versions of an information intervention provided to Africa-American communities regarding colorectal cancer screening (1. Emphasising impact on African-Americans, 2. “Blacks are doing worse than whites”, 3. “Blacks are improving but less than whites”, and 4. Progress – “Blacks are improving over time”). Participants exposed to the two versions highlighting disparities framed in a negative way (2 and 3 above), reported more negative emotional reactions. In contrast the progress framing (4 above), elicited more positive responses and potential participants were more likely to agree to be screened. The results of this trial suggest that the way in which health information is presented can influence attitudes and intentions, with reports about progress yielding a more positive effect on intention. The authors note that this is especially important among those with high levels of medical mistrust. Befort et al. [88] compared the effectiveness of group versus one-on-one telephone counselling for 34 women in hard-to-reach rural areas.

Compliance with the 24-week program was similar between groups, although they did report a significant improvement in weight loss with those in the group-based intervention losing more than those receiving individual counselling. Befort et al. [88] also found the group program to be cost-effective (USD714.43 versus USD1029.06).

##### Other

In a systematic review of diabetes care interventions for socially disadvantaged groups, Glazier et al. [157] noted that the following factors improved both health outcomes, and participation with the intervention: one-on-one interventions, focussing on behaviour-related tasks, providing feedback, and high intensity interventions delivered over a long duration. The review found that interventions which used mainly didactic teaching and focussed on diabetes knowledge were the least successful.

### Attrition and retention

#### Barriers

Twenty six studies [16-18,20,22-24,30,35,39,40,54],[65,66,70-76,80,81,85,96,123] and six reviews [135,139,145,147,148,158] examined barriers to retaining participants in research and strategies for maximising retention rates. The most common barrier reported related to follow-up data collection was difficulty maintaining participant contact. Challenges to maintaining contact included the transient nature of the lives of those in many socioeconomically disadvantaged groups, with phone numbers and addresses changing frequently. Practical barriers often related to their socially disadvantaged status such as transport difficulties, inability to take time away from work to participate, lack of child-care or simply forgetting about the research which is competing with other priorities of daily living [73].

#### Improving retention rates

##### Incentives and gifts

The most commonly trialled strategy for maintaining involvement of participants throughout a research project was the use of incentives and gifts [18,22-24,30,39,40,71-73,76,80],[81,123,158]. Cash incentives have been found to be more effective than non-cash incentives [23], however the use of study branding or logos on non-cash gifts has been reported to be effective in case studies at keeping participants involved [74]. In a study with young Latino women, Lindenberg et al. [80] found that financial cash incentives were considered unacceptable and that vouchers for grocery or department stores were preferred.

Other studies attempted to maintain contact and participations with other gifts such as clothing, birthday and other holiday cards, mugs and personalised follow-up letters [71,73,80], coffee, food or drinks [40]. If travel is required, Woods et al. [73] found that focus group participants suggested transport vouchers and bus tokens as incentives to return to the study [71,73]. Some researchers have noted that these little gifts build trust and relationships between participants and researchers [40,70].

Another two studies of patients receiving treatment for substance abuse found that cash payments did not lead to use of the incentive to purchase illicit drugs and participants reported no perception of coercion [23,24]. In fact the studies found high participant satisfaction with the study, better follow-up rates and reduced tracking efforts.

##### Multiple tracking, reminders and contact procedures

Keeping in regular contact with participants has been reported as an effective method of lowering attrition. Ensuring that researchers have multiple forms of contact for each participant (e.g., phone, mail, email, address and other contact persons), has been reported to be essential for maintaining contact with participants from vulnerable groups who may be highly mobile [17,20,22,30,39,40,54,75],[81,85] as well as having contact details of significant others [20,66,76]. Using a ‘participant-centred approach’ during these multiple contacts, such as personalised telephone calls rather than generic reminder letters enhances retention [18,20,73,123]. In a small pilot study with 48 methamphetamine injectors, Maher et al. [65] found mobile phone text messaging was a successful method of reaching participants (73% contacted) throughout the study. Participants reported that the text messaging was acceptable and there were no differences in the demographics between those who were retained and those who dropped out. Meyers et al. [66] provide a detailed analysis of the tracking strategies they used to obtain extremely high retention rates with substance-abusing youth. The authors described enhanced tracking efforts such as obtaining various contact details during their substance abuse treatment and post treatment, use of a locator form which recorded the contact details of family members, agencies they use and community locations they frequent. Small participant incentives were offered for follow-up (USD5) and approximately 40% of participants required six or more contacts in order to achieve follow-up interview completion. Most (60%) follow-up interviews were conducted in community settings preferred by participants such as fast food outlets. The strategies achieved 94% retention at one month follow-up and 92% retention at six month follow-up. The authors estimated that the additional tracking and retention strategies cost approximately USD85 per participant per follow-up wave.

##### Altruism and benefits of research

Some studies reported appealing to participants’ altruism in terms of the benefits the research might generate to people like themselves [30,40,71,72]. This was often included in participant letters or reinforced during contact with interviewers.

##### Building relationships and trust

Flexibility in scheduling follow-up appointments, responding to messages positively, providing a caring environment, being courteous and frequent contact were all strategies designed to build relationships with participants and reduce attrition [16,30,35,66,70-72,74,76,158]. Flexibility in scheduling appointments addresses practical barriers as well such as difficulties with child care or time from work. Brown et al. [72] reported that participants were more likely to remain in clinical trial research if their doctor helped them feel comfortable. Similarly McMillan et al. [96] credit high retention rates (78% at 6 weeks) of low-income pregnant women on the participants’ midwives involvement.

Establishing a toll-free number where participants can contact researchers free of cost has also been reported [39,54]. Building a project community Signorello et al. [54] used an annual newsletter for longitudinal research participants providing updates on the research. Some studies reported appealing to participants’ altruism in terms of the benefits the research might generate to people like themselves, as well as for them [40,71,72]. This was often included in participant letters or reinforced during contact with interviewers.

##### Comparison studies

Two randomised trials of strategies to minimise attrition were found [23,24]. Festinger et al. [23] randomly assigned participants who were receiving treatment for substance abuse to receive (USD) $70, $100, $130 or $160 incentives in either cash or a gift card for returning for a 6-month follow-up. The trial found that larger cash amounts resulted in the highest follow-up rates and fewer additional tracking calls [24].

##### Multiple strategies

It is worth noting that all studies used multiple strategies to maximise retention, commonly involving at least a comprehensive tracking strategy as well as incentives or gifts (Table 8).

**Table 8:** **Summary of the results from ‘ *retention *’ studies included in the review (n = 26 studies)**

**Barriers**	**Strategies**
Difficulty maintaining contact; highly mobile populations; frequently changing contact numbers	• Implementing tracking procedures with multiple contact methods – i.e., phone, mail, email [17,20,22,30,39,40,54,66],[74,75,81,85].
	• Contact details of significant others [20,30,76,66].
Practical barriers such as transport difficulties, lack of child care, lack of leave from work	• Incentives (cash and other gifts) to study participants as reimbursement for time [18,22,23]*,[24]*,[30,39,40,70-73,76,80,81,123].
	• Participant-centred approach: personalised, tailored individualised approach to follow-up calls or visits [39,54,66,73,96,123] and flexible (accommodating) protocols [16,30,35,66,70,72,74,76] and providing transport or child care [30].
	• Scheduling follow-up assessments to coincide with existing appointments [22,30].
	• Toll-free numbers [39,54].
Forgetting to return for follow-up	• Use of study logos on gifts [74].
	• Phone text message reminders [65].
	• Keep in regular contact [18,22,30,65,67,76,81,118].
	• Highlighting benefits of research during follow-up contact [30,40,71,72].

*Indicates good evidence from randomised controlled trial (see also Table 1).

## Discussion

This is the first literature review to consider all of the research process where representativeness of socially disadvantaged groups may be compromised; sampling, recruitment, data collection, intervention delivery and retention. While previous reviews exist, most are now dated and focussed on one point in the research process, mainly the recruitment or retention of socially disadvantaged groups, and do not consider issues of engagement such as compliance with measurement and intervention. Previous reviews are also limited in scope focusing either on one target group of interest, one type of research study design or one type of intervention. The current review is greater in scope and comprehensiveness providing the reader with a complete picture of strategies for improving socially disadvantaged group participation in research for any given group, within any given research study type and using any given type of intervention strategy. Based on the outcomes of the 116 primary studies included in the current review and 31 literature reviews, a considerable number of barriers to the inclusion of socially disadvantaged groups in health and medical research were identified.

Strategies with good evidence of effectiveness were rare with only nine RCTs identified amongst the 116 studies. Based on this higher level evidence, there was no clear dominant strategy. The trials found mixed results for the effectiveness of incentives to enhance recruitment of socially disadvantaged groups into research [42,43]. Similarly, variations in designs of recruitment letters for socially disadvantaged groups (e.g., hand written or printed [106]; generic or culturally sensitive [43]; and advance letter or no advance letter [36], found mixed results. One trial comparing data collection methods found that telephone interviews resulted in more complete data than mailed health surveys [90]. Two RCTs of strategies for improving intervention fidelity suggest that framing health information in a positive way emphasising progress achieved in the health area [37] and that group-based health counselling achieved greater outcomes than one-on-one counselling for hard-to-reach rural participants [88]. Finally, two trials of cash incentives found that larger cash amounts resulted in higher retention rates [23,24].

A large number of strategies supported by fair and poor research evidence are outlined. One dominant theme was community engagement. Involving community groups and organisations in study design, sampling, recruitment, data collection, and intervention delivery was reported as essential by most studies improving recruitment, participation and retention.

Acknowledging the significance of community involvement in research with disadvantaged groups, the National Health and Medical Research Council (NHMRC) [159] in Australia requires that health research with Aboriginal participants includes substantial and formal community involvement at all levels [113,114,118]. An added benefit of community engagement in health research is the enhanced likelihood of facilitating the translation of research outcomes to policy and practice.

### Quality of the evidence base

Synthesising the literature was made complex by the amount of methodological variation between included studies and the decision to combine a review of both barriers and strategies. Differences in study design and procedures, the nature of the health issue under investigation, settings and the target population groups limited comparisons across studies. A crude ‘levels of evidence’ hierarchy was used (as shown in Table 1), and it must be acknowledged that the usefulness of this approach in examining the research quality is limited. For example, while the RCT presents high level evidence of strategy effectiveness and qualitative research is considered low level evidence of strategy effectiveness, qualitative research, if conducted well, is appropriate research design for gaining an understanding of the barriers to research participation. However, when considering the studies included in the review which examined intervention effectiveness there were too few RCTs to pool data. As a result, this review does not provide conclusive evidence regarding the effectiveness of the discussed strategies. Some papers report case-studies or other descriptions of research which were not hypothesis-driven testing of strategies and not high level evidence of effectiveness. We included these studies as it is not possible to conduct RCTs of some multi-factorial strategies. Instead, these case studies provide valuable insights for researchers endeavouring to include socially disadvantaged groups, so long as the limitation in the quality of evidence is acknowledged. The strength of evidence for many of the strategies reported in this review is largely unknown and further robust experimental trials of those strategies which can be tested are required.

### Implications for research practice

It is imperative that all types of health and medical research employ strategies to increase the representation of socially disadvantaged groups. Strategies will need to be designed and tailored according to different study types and research questions. In some cases, it is more appropriate to target the disadvantaged group of interest in the research. In other examples, like clinical trials of new medicines, it is important that various groups are represented in the overall study sample. It is clear from this review that the barriers to research with socially disadvantaged group are numerous and no one single solution to addressing the barriers exists. To address multiple barriers and challenges, a comprehensive, coordinated, multipronged approach involving many strategies across all stages of research needs to be adopted. This has significant time, cost and data ownership implications. Firstly, a long-term view to conducting research with socially disadvantaged groups is necessary. Developing relationships with communities and community groups, including their involvement in the development of procedures and study resources, and extensive formative research and pilot testing, require a considerable amount of time. Providing collaborating communities and groups with adequate feedback following the conclusion of the study, and in some cases, interventions or treatments, further extends project timelines. These activities ought to be reflected in project timelines and acknowledged by academic institutions employing researchers who conduct studies with socially disadvantaged groups. This long-term view should also be extended to publication of research with socially disadvantaged groups.

Secondly, many of the strategies are resource intensive and entail costs which may be additional to the usual costs of managing a research project. The addition of translation services or bilingual research staff, flexibility regarding data collection locations or times, gifts and incentives, culturally tailored resources and materials, extensive participant tracking procedures and additional staff training require consideration of costs. Some strategies such as including all family members in a study to avoid random selection [113], or offering those randomised to control conditions other types of interventions or treatments [148], may increase study costs significantly. These costs can be justified in funding applications and need to be recognised by funding organisations. Finally, in some cases of community collaborative research, data ownership may need to be negotiated and joint authorship with non-academic collaborators included. Academic researchers need to be supported by their academic institutions and scientific journals for publishing collaborative research which may take longer to produce due to authorship negotiations.

The establishment of research centres or research collaborations dedicated to high quality health and medical research with socially disadvantaged groups is one model for addressing many of the issues raised in this review in a coordinated manner [160,161]. Numerous benefits would flow-on from these research centres including pooling of funding and resourcing, drawing on multidisciplinary expertise, promoting a high-level research culture in the field, expansion in the development of partnership links and networks with community organisations and groups which would increase access and recruitment, and training and building the capacity of future leaders in health research with socially disadvantaged groups.

Furthermore, a research collaborative could initiate and maintain a research participant registry for improved access to participants from numerous socially disadvantaged groups [25].

This review found a number of strategies and methodologies that may have a negative impact on health inequities. For example, the use of incentives, which have considerable evidence for improving recruitment and retention in the general literature [162], have negative connotations in some cultures [80]. Also, some commentators have raised ethical concerns regarding the use of incentives with research participants who may be financially compromised [163]. Festinger et al. [23,24] however, provided compelling evidence in their trials of cash incentives with people in substance abuse treatment; participants in these trials did not use the cash incentive to purchase illicit substances and did not perceive the incentives as coercive highlighting the need to test strategies with target groups for both positive and negative effects. Similarly, the way language is used and information framed [37] can have a negative impact on the reactions of some groups to health research. It is difficult to generalise results from one disadvantaged group to another as different cultural factors may be influencing reactions and outcomes.

### Implications for health policy and services

Knowledge gained from health research, particularly health services research, is used to inform the development or improvement of health policy and health care. If a diversity of the population of health service users is not included, service delivery is likely to be inequitable.

Doherty et al. [164] have identified three main hard-to-reach groups within service involvement: 1) the under-represented; 2) the invisible/overlooked; and 3) the service resistant. Reports suggest that the same types of barriers that result in underrepresentation in health research also lead to under-representation in health service use – distrust of authorities, health literacy and communication difficulties and pragmatic financial, transport or employment related barriers [165]. An improvement in the representation of vulnerable groups in health research, particularly health services research, is likely to yield benefits through more equitable service delivery and engagement.

Tudor-Hart first showed that the individual requiring the greatest effort to attend a health service is also the one with the greatest need [166]. While involving socially disadvantaged groups in activities may be resource-intensive and challenging, it has been argued that where sufficient funds allow the development of more creative approaches, it should be possible to engage with all members of society [167]. The strategies described in this review as potentially effective at increasing involvement in health research, may be generalizable to improving access and use of health services.

### Study strengths and limitations

The main limitation of the study is the extent of methodological variation in the studies included in the review. The extent of heterogeneity between studies prohibited combining the results statistically in a meta-analysis. The inclusion of low level evidence also prohibits conclusive comments regarding the effectiveness of many of the strategies discussed in this review. However, this could also be considered a study strength as well; few RCTs were identified to provide a complete assessment of the effectiveness of strategies and inclusion of descriptive studies and case-studies provides valuable insights into ways to increase research with socially disadvantaged groups worth further testing. Finally, the majority of studies included in the review were conducted in developed countries and may not be generalizable to other countries.

## Conclusion

Representativeness can be threatened at various stages of the research process, and researchers must remain mindful of whom they may be excluding in the design and implementation of research studies, and employ strategies to avoid this happening.

Researchers are in a powerful position to influence inequities in health outcomes. Generation of research findings that are representative of all social groups will allow development of an evidence base that can be used by service providers and policy makers to deliver programs and policies that reduce health inequities. Research funding agencies also play a role in ensuring that they fund research that demonstrates consideration of representativeness in its design, budget and timelines given this type of research is likely to be more complex, costly and time-consuming. However as the World Health Organisation’s Commission on the Social Determinants of Health notes the increased investment is highly worthwhile as addressing health inequities will result in health, social and economic benefits for all of society [10].

## Competing interests

The authors declare that they have no competing interests.

## Authors’ contributions

All authors conceived the study. MR conducted electronic searches. BB and MR coded articles. All authors contributed to drafting and finalising the manuscript. All authors read and approved the final manuscript.

## Pre-publication history

The pre-publication history for this paper can be accessed here:

http://www.biomedcentral.com/1471-2288/14/42/prepub
